# Prevalence and determinants of electrocardiographic abnormalities in sub-Saharan African individuals with type 2 diabetes

**DOI:** 10.5830/CVJA-2012-054

**Published:** 2012-11

**Authors:** Anastase Dzudie, Simeon-Pierre Choukem, Félicité Kamdem, Solange Doualla, Henry A Joko, Marielle EE Lobe, Yves M Mbouende, Henry Luma, Abdoul K Adam, Andre P Kengne, Patricia Gouking, Mesmin Dehayem, Jean-Claude Mbanya, Samuel Kingue

**Affiliations:** Department of Internal Medicine, Douala General Hospital, Cameroon; Department of Internal Medicine, Buea Faculty of Health Sciences, Cameroon; Department of Internal Medicine, Douala General Hospital, Cameroon; Department of Internal Medicine, Buea Faculty of Health Sciences, Cameroon; Department of Internal Medicine, Douala General Hospital, Cameroon; Department of Internal Medicine, Douala General Hospital, Cameroon; Department of Internal Medicine, Yaoundé Faculty of Medicine, Cameroon; Department of Internal Medicine, Douala General Hospital, Cameroon; Department of Internal Medicine, Douala General Hospital, Cameroon; Department of Internal Medicine, Douala General Hospital, Cameroon; Department of Internal Medicine, Douala General Hospital, Cameroon; Department of Internal Medicine, Yaoundé Faculty of Medicine, Cameroon; Universite Université des Montagnes, Bangangte, Cameroon; Department of Medicine, University of Cape Town and Medical Research Council, Cape Town, South Africa; Diabetes and Endocrine Service, Yaoundé Central Hospital and Faculty of Medicine, Cameroon; Diabetes and Endocrine Service, Yaoundé Central Hospital and Faculty of Medicine, Cameroon; Diabetes and Endocrine Service, Yaoundé Central Hospital and Faculty of Medicine, Cameroon; Department of Internal Medicine, Yaoundé Faculty of Medicine, Cameroon; Department of Internal Medicine, Yaoundé Faculty of Medicine, Cameroon

**Keywords:** diabetes mellitus, sub-Saharan Africa, Cameroon, ECG, cardiovascular disease

## Abstract

**Aim:**

This study assessed the prevalence and determinants of electrocardiographic abnormalities in a group of type 2 diabetes patients recruited from two referral centres in Cameroon.

**Methods:**

A total of 420 patients (49% men) receiving chronic diabetes care at the Douala General and Yaoundé Central hospitals were included. Electrocardiographic abnormalities were investigated, identified and related to potential determinants, with logistic regressions.

**Results:**

The mean age and median duration of diagnosis were 56.7 years and four years, respectively. The main electrocardiographic aberrations (prevalence %) were: T-wave abnormalities (20.9%), Cornell product left ventricular hypertrophy (16.4%), arrhythmia (16.2%), ischaemic heart disease (13.6%), conduction defects (11.9%), QTc prolongation (10.2%) and ectopic beats (4.8%). Blood pressure variables were consistently associated with all electrocardiographic abnormalities. Diabetes-specific factors were associated with some abnormalities only.

**Conclusions:**

Electrocardiographic aberrations in this population were dominated by repolarisation, conduction defects and left ventricular hypertrophy, and were more related to blood pressure than diabetes-specific factors.

## Abstract

A major threat to the health of diabetes subjects is cardiovascular disease (CVD), which currently accounts for about three-quarters of all deaths in diabetes patients in major populations and settings.^1^ Attempts to maintain cardiovascular health in diabetics include: (1) routine prescription of medications with proven beneficial effects on cardiovascular health, such as statins and aspirin; (2) investigation and treatment of individuals with abnormal levels of modifiable risk factors; (3) monitoring of individuals for infra-clinical changes, which are indicators of future high risk for cardiovascular events, or those with less-advanced stages of diabetes, whose course could be modified through early intervention.^2^

The electrocardiogram (ECG) is widely used for monitoring.^3^ ECG changes appear early in the course of diabetes, and usually include alterations such as sinus tachycardia, QTc prolongation, QT dispersion, changes in heart rate variability, ST–T changes, and left ventricular hypertrophy. These changes and others, detected with the use of a resting ECG, often together with an exercise ECG, are used to detect silent ischaemia, assess prognosis and predict future risk. Because the ECG is a non-invasive and relatively easy test to perform, it is used in the series of investigations conducted as part of the annual clinical evaluation of people with diabetes around the world.^3^

The use of this modality however varies substantially, guided essentially by the availability of ECG machines and the cost of such investigations. As a result, the regional office of the International Diabetes Federation (IDF) for Africa recommends ECG monitoring in diabetes only at the secondary or tertiary level of the healthcare system where facilities for performing an ECG are more readily available.^4^

Therefore in sub-Saharan Africa, the majority of patients with diabetes who receive care in primary healthcare facilities do not have routine ECG screening. Failure to perform regular ECGs means that opportunities to improve cardiovascular health in this population are being missed. Furthermore, our knowledge of the major ECG abnormalities and their determinants in this environment remains very limited.

In this study we assessed the distribution of ECG aberrations and investigated their potential determinants in a group of individuals with type 2 diabetes who were receiving chronic care in two referral hospitals in the two largest cities of Cameroon, Central Africa.

## Methods

The out-patient sections of the Yaounde Central Hospital’s diabetes and endocrine service, and the Douala General Hospital’s (DGH) internal medicine service and sub-specialties served as settings for recruitment of participants for this study. The Yaounde Central Hospital (YCH) has been described in detail elsewhere.^5^,^6^ The DGH internal medicine and sub-specialities service has an individualised, dedicated endocrine section, which is the main referral centre for endocrine diseases and diabetes in Douala, the second major city of Cameroon (approximately 2.5 million people). Patients with diabetes and its complications, residing in Douala and surrounding regions were the most likely to receive care in our clinic during the study period.

Overall, the healthcare system in Cameroon is organised into primary, secondary and tertiary levels. Care at the primary level is provided by nurses and general practitioners and is essentially geared towards acute conditions. Secondary-level facilities provide access to some form of specialist care. Tertiary-level facilities (including YCH and DGH) serve as a referral hospital for primary- and secondary-level health facilities, and for routine consultations and follow up, as in our study.

From January 2010, the Yaounde health service has had three endocrinologists and the Douala health service two. Patients with diabetes who received chronic care in the two study clinics were required to have an annual evaluation as part of their routine care. In addition to a clinical consultation, this evaluation included: (1) an assessment of diabetes control (fasting glucose and haemoglobin A_1c_ levels); (2) an assessment of chronic complications (eyes: fundoscopy, kidney function: albuminuria, serum urea and creatinine levels); (3) a cardiovascular work up including an assessment of lipid profiles (total cholesterol, high-density lipoprotein cholesterol and triglycerides) and a resting ECG.

Participants in this study were recruited from patients presenting for these annual evaluations. The study was approved by the administrative authorities of the two health facilities, and ethical clearance was obtained from the Cameroon National Ethics Committee.

Four hundred and twenty individuals with type 2 diabetes receiving chronic care in the two study facilities were consecutively enrolled over a two-year period from January 2008 to January 2010. Only the patients’ first consultation during this period was considered, and no other exclusion criteria were applied. The type of diabetes was based on the diagnosis of the attending physician. In addition, patients had to be at least 30 years of age at the time of their first diagnosis of diabetes.

Blood pressure (mmHg) was measured on the right arm with the participant in a seated position, after 10 minutes’ rest, with an Omron® MX2 basic electronic device (Omron Healthcare Co, Ltd, Kyoto, Japan) with the appropriate cuff size. The average of two measurements recorded five minutes apart was used in this study. Body weight (kg) was measured in light clothing, using a SECA® scale, and height (m) was measured with a standard stadiometer.

The body mass index (BMI) for each patient was calculated as weight/height^2^ (kg/m^2^). The waist circumference (cm) was measured with a tape measure on the horizontal plane midway between the lowest rib margin and upper edge of the iliac crest.

A 12-lead resting ECG was done on all subjects using the Cardi Max Fx-7302®. All ECG tracings were centrally interpreted by the same investigator who is a cardiologist (AD) and did not know the subjects’ backgrounds. Significant ECG findings such as ST-segment elevation or depression, T-wave aberrations (inversion or tall T wave), bundle branch block, left ventricular hypertrophy (LVH), right and left atrial enlargement, arrhythmias and other changes were noted.

LVH was defined according to three different criteria:

• Cornell voltage-duration product [(RaVL + SV3) × QRS complex duration] > 2.623 mm × ms in men and > 1.558.7 mm × ms in women,^7^
• Cornell voltage (SV3 + RaVL > 24 mm in women and 28 mm in men)
• Sokolov-Lyon index (SV1 + RV5/6 < 35 mm). Compared with echocardiography, the cut-off values for the Cornell voltage duration product gave the best sensitivity with a specificity of 95%.^7^

ECG measurements were done with a ruler on the resting ECG tracings, and were expressed as the average of three determinations on consecutive QRS complexes. R-wave amplitude in aVL and S-wave depth in V3 were measured as the distance (mm) from the isoelectric line of their zenith and nadir, respectively. QRS duration was measured from the beginning to the end of the QRS complex. QTc prolongation was defined as a QTc > 460 ms in both men and women.

A diagnosis of ischaemic heart disease was made based on the American Heart Association criteria. These criteria include ECG features of significant ST-segment depression, defined as an ST-segment depression > 1 mm in more than one lead, and T-wave inversion. Myocardial infarction was defined as an ST-segment elevation (convex upwards) > 0.08 s, associated with T-wave inversion in multiple leads, and reciprocal ST-segment depression in opposite leads.

## Statistical analysis

Data were analysed using SPSSR version 17 for Windows (SPSS, Chicago, IL). Differences in means and proportions for participants’ characteristics were assessed using analysis of variance and χ^2^ tests as applicable, and the influence of likely confounders was adjusted for with logistic regressions models. A probability of *p* < 0.05 was set as the threshold of statistical significance.

## Results

Of the 420 patients recruited, 207 (49%) were men and 250 (56%) were from the Yaounde centre. The mean age was 56.7 years and the median duration of diagnosed diabetes was four years (IQR 25th to 75th percentiles: 1–9).

As expected, anthropometric characteristics were different between men and women. Diabetes control was also poorer in men than in women (all *p* < 0.04), otherwise men were similar to women with regard to many other characteristics, including history of diabetes, treatment and complications, and cardiovascular risk profile (Table 1).

**Table 1.** Profile Of The 420 Men And Women With Type 2 Diabetes

*Variables*	*Men n (%)*	*Women n (%)*	p	*Total n (%)*
Number (%)	207 (49)	213 (51)		56.7 (9.92)
Age (years)	55.9 (9.83)	57.5 (9.96)	0.09	4 (1–9)
Median (range) known duration of diabetes (years)	4 (0–9)	4 (1–8)	0.71	213 (50.7)
Parental history of diabetes	103 (49.7)	110 (51.6)	0.69	32 (7.6)
Smoking	27 (13.1)	5 (2.3)	< 0.001	28.5 (5.2)
Body mass index (kg/m^2^)	27.2 (4)	29.7 (6)	< 0.001	95.1 (11.9)
Waist circumference (cm)	95.3 (10.8)	94.9 (12.92)	0.71	101.2 (11.8)
Hip circumference (cm)	98.5 (10)	103.7 (12.9)	< 0.001	0.94 (0.10)
Waist-to-hip ratio	0.96 (0.08)	0.91 (0.11)	< 0.001	142.2 (25.3)
Hypertension and treatments				
Systolic blood pressure (mmHg)	142.8 (23.6)	141.6 (26.91)	0.61	85.1 (13.2)
Diastolic blood pressure (mmHg)	85.6 (12.2)	84.5 (14.15)	0.37	57.1 (18.2)
Pulse pressure (mmHg)	57.2 (16.8)	57.1 (19.49)	0.95	211 (50.2)
Hypertension	97 (46.8)	114 (53.5%)	0.17	186 (44.3)
Any blood pressure-lowering medication	83 (40.1)	103 (48.4)	0.09	139 (33.1)
ACE inhibitors	70 (33.8)	69 (32.4)	0.84	5 (1.2)
ARA II antagonists	2 (1)	3 (1.4)	0.99	118 (28.1)
Diuretics	54 (26.1)	64 (30)	0.37	69 (16.4)
Calcium channel blockers	33 (15.9)	36 (16.9)	0.79	30 (7.1)
Beta-blockers	7 (3.4)	23 (10.8)	0.004	185 (49)
Lipid profile and lipid-modifying therapies				
Total cholesterol (mg/dl)	187 (49)	184 (51)	0.57	47 (18)
HDL cholesterol (mg/dl)	47 (19)	48 (18)	0.52	101 (67–141)
Median (range) triglycerides (mg/dl)	99 (64–142)	102 (68–140)	0.62	35 (13.2)
Lipid modifying therapies	19 (9.2)	16 (7.5)	0.58	1 (0.2)
History of cardiovascular disease				
Coronary heart disease	0 (0.0)	1 (0.5%)	0.32	15 (3.6)
Cerebrovascular diseases	6 (2.9)	9 (4.2%)	0.46	6 (1.4)
Lower limb occlusive vascular disease	3 (1.4)	3 (1.4%)	0.97	89 (67–111)
Median (range) creatinine clearance (ml/min/1.73 m^2^)	91 (70–113)	88(63–108)	0.23	273 (66)
Diabetes treatment and control				
Metformin	133 (64.7)	143 (67%)	0.58	185 (44)
Suphonamide	93 (45)	92 (43%)	0.69	9 (2.1)
Acarbose	2 (0.9)	7 (3.3%)	0.19	68 (16.2)
Insulin	37 (17.9)	31 (14.5%)	0.34	177 (81)
Fasting capillary glucose (mg/dl)	185 (85)	169 (77)	0.04	8.2 (2.3)
Haemoglobin A_1c_ (%)	8.5 (2.3)	7.9 (2.2)	0.03	
Microvascular complications				
Any diabetic retinopathy	38 (18.3%)	28 (13.1)	0.14	66 (15.7)
Any diabetic nephropathy	30 (14.5%)	37 (17.4)	0.42	67 (15.9)
Any diabetic neuropathy	52 (25.1%)	42 (19.7)	0.18	94 (22.4)

With few exceptions, participants’ characteristics were mostly similar across the participating centres. The few exceptions related to hip circumference (*p* < 0.001), diastolic blood pressure (*p* < 0.001), haemoglobin A1c level (*p* < 0.001), creatinine clearance rate (*p* = 0.04), the use of ACE inhibitors (*p* = 0.01) and the presence of neuropathy (*p* = 0.008).

**Fig. 1.**
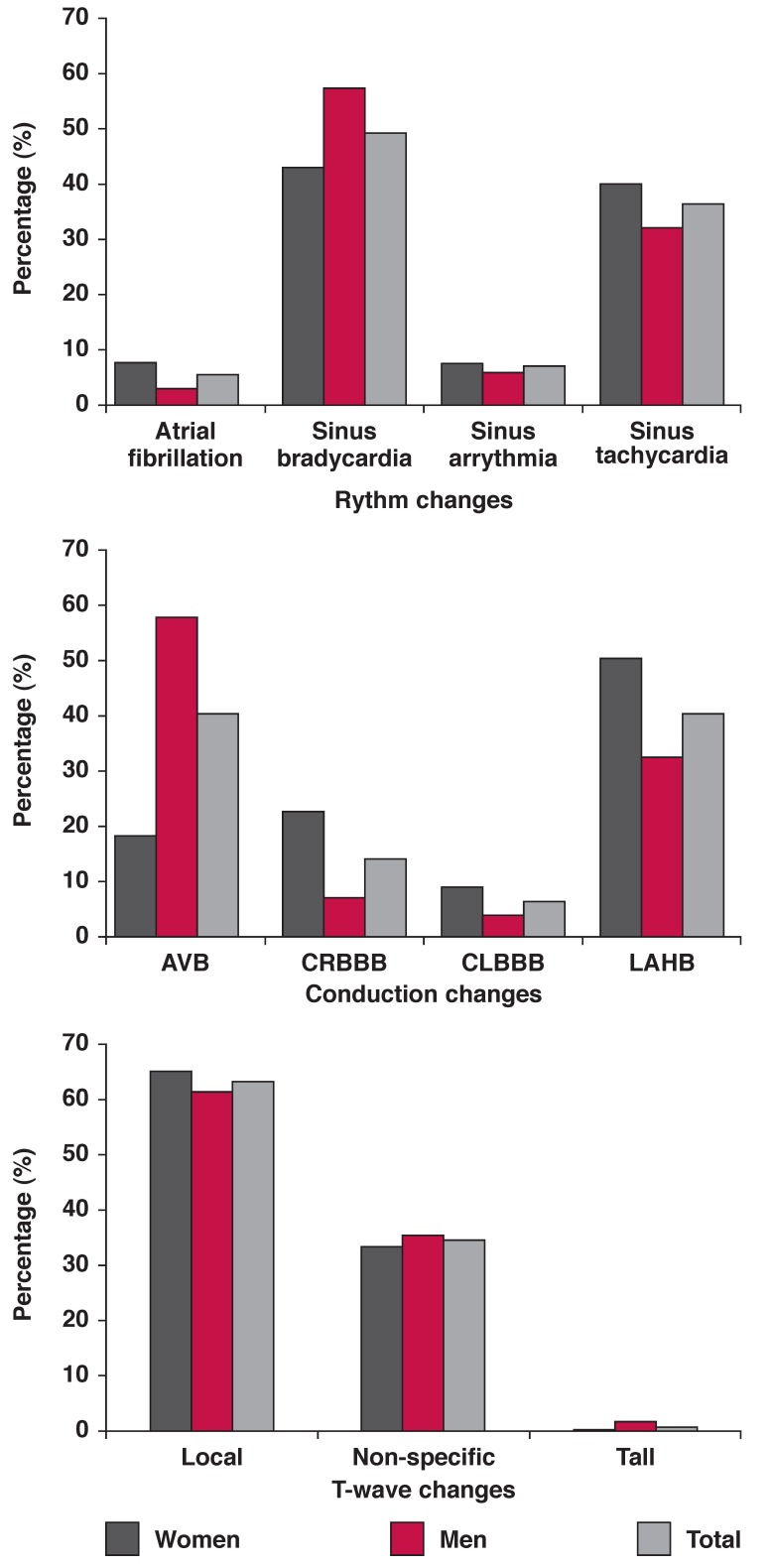
Rhythm, conduction and T-wave changes in 420 men and women with type 2 diabetes.

The distribution of ECG abnormalities was: T-wave aberrations (20.9%), left ventricular hypertrophy according to the Cornell product criteria (16.4%), arrhythmia (16.2%), ischaemic heart disease (13.6%), conduction defects (11.9%), QTc prolongation (10.2%) and ectopic beats (4.8%). Unlike T-wave aberrations and left ventricular hypertrophy, the prevalence of major aberrations was similar in men and women (Table 2). The distribution of subtypes of arrhythmia, conduction defects and T-wave aberrations is shown in Fig. 1.

**Table 2.** ECG Changes In 420 Men And Women With Type 2 Diabetes

*Variables*	*Men n (%)*	*Women n (%)*	p	*Total n (%)*
Number (%)	207 (49)	213 (51)		420
Arrhythmia	31 (15)	37 (17.4)	0.51	68 (16.2)
Conduction changes	28 (13.5)	22 (10.3)	0.37	50 (11.9)
Ectopic beats	10 (4.8)	10 (4.7)	0.99	20 (4.8)
T-waves changes	53 (25.6)	35 (16.4)	0.02	88 (20.9)
QTc prolongation	18 (8.7)	25 (11.7)	0.34	43 (10.2)
Ischaemic heart disease	34 (16.4)	23 (10.8)	0.12	57 (13.6)
Left ventricular hypertrophy by diagnostic criteria				
Cornell product	14 (6.7)	55 (25,8)	< 0.001	69 (16.4)
Sokolov index	17 (8.2)	7 (3.3)	0.03	24 (5.7)
Cornell index	12 (5.8)	5 (2.3)	0.09	17 (4.1)

The distribution of subtypes of conduction defects was significantly different in men and women (*p* = 0.03). Significant predictors of ECG abnormalities are shown in Table 3. Age variables (age at diabetes diagnosis and duration of diagnosed diabetes), and blood pressure variables were the common significant predictors of ECG abnormalities.

**Table 3.** Odds Ratio And 95% Confidence Intervals For Predictors Of ECG Changes

*Variables*	*Arrhythmia*	*Conduction*	*T-wave changes*	*Long QTc*	*IHD*	*LVH*	*Ectopic beat*
Age at diabetes diagnosis (years)	1.02 (0.99–1.04)	1.06 (1.02–1.09)*	1.02 (0.99–1.04)	1.02 (0.99–1.06)	1.00 (0.97–1.03)	1.05 (1.02–1.08)*	1.06 (1.01–1.12)*
Duration of diagnosed diabetes (years)	1.02 (0.97–1.06)	1.01 (0.96–1.07)	1.04 (1.00–1.08)*	1.08 (1.03–1.13)*	1.02 (0.98–1.07)	1.05 (1.00–1.10)*	1.04 (0.97–1.12)
Gender (men vs women)	1.16 (0.69–1.96)	0.66 (0.36–1.22)	0.55 (0.34–0.89)*	1.40 (0.73–2.68)	0.62 (0.35–1.09)	4.86 (2.54–9.25)*	0.84 (0.34–2.12)
Recruitment centre (Yaoundé vs Douala)	0.89 (0.52–1.53)	0.89 (0.48–1.66)	1.78 (1.10–2.87)	1.05 (0.55–2.02)	1.28 (0.73–2.26)	3.79 (2.13–6.75)*	2.36 (0.93–5.95)
Presence/history of nephropathy	0.69 (0.34–1.38)	0.76 (0.33–1.73)	0.45 (0.24–0.83)*	0.53 (0.25–1.15)	0.47 (0.23–0.95)*	0.66 (0.31–1.40)	0.52 (0.17–1.66)
Metformin use	1.06 (0.61–1.84)	0.87 (0.46–1.67)	1.04 (0.63–1.73)	1.86 (0.97–3.55)	0.85 (0.46–1.56)	0.89 (0.48–1.64)	0.47 (0.15–1.46)
Suphonylurea use	0.87 (0.51–1.47)	0.90 (0.49–1.65)	0.71 (0.44–1.15)	1.47 (0.76–2.86)	0.58 (0.33–1.02)	1.23 (0.69–1.20)	0.62 (0.25–1.57)
Insulin use	0.60 (0.31–1.18)	3.26 (0.96–11.09)	1.06 (0.54–2.09)	0.51 (0.26–1.11)	1.11 (0.50–2.47)	0.93 (0.40–2.17)	1.44 (0.31–6.75)
Waist circumference (cm)	0.98 (0.96–1.00)	1.01 (0.98–1.03)	0.98 (0.96–1.00)	1.03 (1.01–1.06)*	1.00 (0.97–1.02)	1.02 (1.00–1.04)	1.01 (0.97–1.04)
Systolic blood pressure (mmHg)	1.00 (0.99–1.01)	1.01 (1.00–1.03)*	1.01 (1.00–1.02)*	1.02 (1.01–1.03)*	1.01 (0.99–1.02)	1.02 (1.01–1.03)	1.01 (0.99–1.02)
Diastolic blood pressure (mmHg)	1.00 (0.98–1.02)	1.01 (0.99–1.04)	1.01 (0.99–1.03)	1.05 (1.02–1.07)*	1.01 (0.99–1.03)	1.01 (0.99–1.04)	1.01 (0.98–1.05)
Pulse pressure (mmHg)	1.01 (0.99–1.02	1.02 (1.00–1.04)*	1.02 (1.00–1.03)	1.02 (1.00–1.03)	1.01 (0.99–1.03)	1.03 (1.01–1.05)*	1.00 (0.98–1.03)
Heart rate (beats/min)	1.01 (0.99–1.03)	0.98 (0.96–1.01)	0.98 (0.96–1.00)*	1.05 (1.03–1.08)*	0.99 (0.97–1.01)	0.99 (0.97–1.01)	1.03 (0.97–1.04)
Total cholesterol (mg/dl)	0.60 (0.35–1.04)	1.15 (0.63–2.10)	1.55 (0.96–2.52)	1.22 (0.64–2.36)	1.27 (0.72–2.24)	1.24 (0.71–2.16)	1.19 (0.48–2.97)
HDL cholesterol (mg/dl)	0.66 (0.15–2.82)	1.39 (0.29–6.51)	2.23 (0.63–7.98)	1.03 (0.17–6.01)	3.82 (0.92–15.96)	1.13 (0.24–2.39)	1.97 (0.19–19.98)

*p < 0.05; IHD, ischaemic heart disease; LVH, left ventricular hypertrophy; all models are adjusted for gender, age and diabetes diagnosis, known duration of diabetes and study centre

The presence of diabetic nephropathy was significantly associated with T-wave aberrations [OR: 0.45 (95% CI: 0.24–0.83)] and ischaemic heart disease [OR: 0.47 (0.23–0.95)]; otherwise, diabetes medications and markers of disease control were not associated with the outcomes. Waist circumference was associated with a 3% (95% CI: 1–6%) higher risk of QTc prolongation, otherwise no other marker of adiposity was associated with the outcomes. Similarly, none of the lipid variables was significantly associated with ECG abnormalities.

## Discussion

This study revealed the high prevalence of ECG aberrations in this population of individuals with a short duration of clinically overt type 2 diabetes. While some of these aberrations were benign, others were potential indicators of the presence of serious conditions such as ischaemic heart disease, or were associated with increased future risk of fatal and non-fatal cardiovascular events. The minimal use of preventive treatment for cardiovascular disease in this population highlights the scope for improving cardiovascular health in people with type 2 diabetes in this region.

Some aspects of ECG abnormalities in people with diabetes, such as those relating to LVH,^8^ ischaemic heart disease^9^ or QTc prolongation^10^ have been investigated in a few studies on diabetics in Africa. To the best of our knowledge, however, there is no recent study that has investigated the full spectrum of resting ECG aberrations and potential determinants in people with diabetes in this part of the world.

In accordance with a previous study in Tanzania,^8^ we found a 16% prevalence of LVH in our study. Interestingly, blood pressure variables were also the main determinants of LVH, with approximately similar range of effects.^8^

That more than one in 10 participants in the current study had ECG aberrations suggestive of ischaemic heart disease has relevance in sub-Saharan Africa where cardiovascular diseases are not considered a major priority health issue in people with diabetes.^11^ In a previous study in the same region, using both resting and exercise ECGs, a prevalence of 7.5% for cardiac ischaemia was found; although this was based on a small sample size.^12^

Even after accounting for the uncertainties around the estimates from this and other studies in sub-Saharan Africa,^9^ our findings support a growing prevalence of ECG-diagnosed ischaemic heart disease in diabetes patients in our region over time. This prevalence was similar to that found in stroke survivors in Africa,^13^ and therefore provides more evidence in support of the high cardiovascular risk of diabetes patients in this part of the world.

It is possible that the prevalence of ECG-diagnosed cardiac ischaemia was inflated in our study for at least two reasons: (1) in the absence of a correlation between ECG aberrations and clinical features, some of the observed ST-segment and T-wave changes could have been variants of normal ECGs, as previously described in blacks;^14^ (2) some of the repolarisation changes could have been secondary to hypertension, which is very common in diabetes patients in this region.^5^

In a cohort of black and white subjects with no known cardiovascular disease who were participants of the Health, Aging, and Body Composition study (Health ABC study), the presence of major or minor ECG aberrations at baseline was associated with coronary heart disease risk during follow up, independent of classical cardiovascular risk factors.^15^ The findings of the Health ABC study suggest that the presence of ECG aberrations, including those used to diagnose cardiac ischaemia in our study, should be given consideration as they may indicate an adverse underlying cardiovascular risk profile.

Approximately 13% of participants in this study were on a statin, preventive treatment widely recommended for routine use in people with diabetes. No correlation was found between statin use and ECG-diagnosed ischaemic heart disease. This suggests that the use of statins in this population could be almost doubled by using ECG criteria to diagnose for ischaemic heart disease. It was shown in a recent study that the use of recommended preventive therapies for cardiovascular disease risk reduction, based on global risk evaluation, was limited in Africa in people with diabetes and those without.^16^

Our study had some limitations. In the absence of follow up, we were unable to establish any causal relationship between identified predictors of cardiovascular risk and ECG aberrations. This was a hospital-based study and therefore included participants who may not have been typical of those in the community where the majority of type 2 diabetes persons remain undiagnosed.^17^ While this could have affected the prevalence of ECG changes found in our study, it was less likely to have affected the direction of associations described, and therefore would not have invalidated the major findings from this study.

That ECGs were interpreted by an investigator who was unaware of the clinical background of the patients, which could have affected the prevalence of some of the outcomes. Indeed, using such an approach resulted at best in a description of significant changes, with no assumption about possible correlations between coincident aberrations in the same patient.

Our study had some major advantages, including the considerable sample size, which gave us reasonable statistical power to reliably investigate the parameters. We were also able to investigate the full spectrum of resting ECG aberrations, which no previous study has achieved in Africa. The extensive data collection of both clinical and biological profiles enabled a wide range of predictors to be investigated for their possible link with prevalent ECG aberrations.

## Conclusion

ECG aberrations are frequent in people with diabetes in sub-Saharan Africa. While some may be benign, others are indicators of serious underlying conditions or high future risk for cardiovascular disease. These aberrations have the potential to improve cardiovascular disease risk stratification and the implementation of preventative strategies in people with diabetes in sub-Saharan Africa.

The growing prevalence of serious ECG aberrations over time suggests the need for strategies to monitor such changes and their determinants, so as to refine the cardiovascular preventative strategies in sub-Saharan Africa. Elsewhere, dedicated diabetes registries have successfully served these functions.
